# Boric acid induces cytoplasmic stress granule formation, eIF2α phosphorylation, and ATF4 in prostate DU-145 cells

**DOI:** 10.1007/s10534-014-9809-5

**Published:** 2014-11-26

**Authors:** Kimberly A. Henderson, Sarah E. Kobylewski, Kristin E. Yamada, Curtis D. Eckhert

**Affiliations:** 1 Interdepartmental Program in Molecular Toxicology, University of California, Los Angeles, CA USA; 2 Department of Environmental Health Sciences, University of California, Charles E. Young Drive, Los Angeles, CA 90095 USA

**Keywords:** Boron, Boric acid, eIF2α, ATF4, DU-145 cells

## Abstract

Dietary boron intake is associated with reduced prostate and lung cancer risk and increased bone mass. Boron is absorbed and circulated as boric acid (BA) and at physiological concentrations is a reversible competitive inhibitor of cyclic ADP ribose, the endogenous agonist of the ryanodine receptor calcium (Ca^+2^) channel, and lowers endoplasmic reticulum (ER) [Ca^2+^]. Low ER [Ca^2+^] has been reported to induce ER stress and activate the eIF2α/ATF4 pathway. Here we report that treatment of DU-145 prostate cells with physiological levels of BA induces ER stress with the formation of stress granules and mild activation of eIF2α, GRP78/BiP, and ATF4. Mild activation of eIF2α and its downstream transcription factor, ATF4, enables cells to reconfigure gene expression to manage stress conditions and mild activation of ATF4 is also required for the differentiation of osteoblast cells. Our results using physiological levels of boric acid identify the eIF2α/ATF pathway as a plausible mode of action that underpins the reported health effects of dietary boron.

## Introduction

Human boron intake is affected by both regional geology, which contributes to its concentration in drinking water and agricultural soils, and by local preferences for boron rich fruits, vegetables, nuts, legumes and plant based beverages such as coffee, wine and fruit juice (Rainey and Nyquist 1998; Barranco et al. 2007a, b). Higher intake of boron is associated with a lower risk of prostate cancer and volume in men, lung cancer in women, and bone mass in animals (Mahabir et al. 2008; Korkmaz et al. 2007). In humans, 83–98 % of dietary boron is excreted in urine in 96 h and is distributed as boric acid (BA) to all tissues (WHO 1998). Cellular studies at physiological concentrations of BA were made possible by the development of a procedure to deplete BA from cell culture media (Bennett et al. 1999). In healthy men blood BA concentrations range from below a detectable limit of 4.7 µM B (48.5 µg B/g wet blood) in low boron regions to 350 µM B (3,600 µg B/g wet blood) in boron mine workers (Bolt et al. 2012). BA is also a developmental and reproductive toxin with a lowest observed adverse effect level (LOAEL) for testicular damage in rats reported at 620 µM B (Ku et al. 1993; Chapin et al. 1997). The U.S. Environmental Protection Agency set a reference dose (RfD) at 0.2 mg B/kg/d (EPA 2004). BA inhibits the proliferation of LNCaP and DU-145 prostate cancer cells in a dose-dependent manner within the physiological range (Barranco and Eckhert 2004). To identify a functional molecular target of BA in the absence of a stable radioactive boron isotope we based our approach on the knowledge that BA interacts with NAD^+^ (Johnson and Smith 1976; Kim et al. 2003, 2004). CD38, a multifunctional enzyme in the plasma membrane, converts extracellular NAD^+^ into cyclic ADP ribose (cADPR), the only known agonist of the ryanodine receptor calcium (Ca^2+^) channel. We identified cADPR as a molecular target of BA and demonstrated that physiological levels of BA inhibit cADPR-activated ER Ca^2+^ release within seconds of treatment and reduce ER luminal Ca^+2^ concentrations ([Ca^+2^]_ER_) (Henderson et al. 2009; Kim et al. 2006).

High [Ca^+2^]_ER_ is required for protein folding in the ER and provides a steep gradient between the ER (100–500 µM) and cytoplasm (20–100 nM) that enables rapid Ca^+2^ signaling (Berridge 1997, 2002). Treatment with thapsigargin, a plant sesquiterpene lactone, inhibits the ATP-dependent ER Ca^+2^ pump (SERCA) resulting in lower [Ca^+2^]_ER_ and accumulation of unfolded and misfolded proteins (Conn 2011; Deniaud et al. 2008). Unfolded proteins sequester glucose-regulated protein (GRP78/BiP) away from the luminal domain of the ER transmembrane protein, double stranded RNA-activated protein kinase (PKR)-like ER kinase (PERK) (Hendershot 2004; Shen et al. 2004). The release of GRP78/BiP enables PERK to dimerize and phosphorylate the serine 51 residue of eukaryotic initiation factor 2α (eIF2α). Phosphorylation of eIF2α mediates the integrated stress response (ISR) which maintains cellular homeostasis under mild ER stress and redirects transcription and translation to a subset of proteins that include the transcription factor ATF4 which is usually down-regulated in models of human prostate cancer (So et al. 2009). Non-toxic levels of BA have been reported to activate phosphorylation of eIF2α in mammalian cells (Henderson and Eckhert 2006; Henderson 2009) and teratogenic levels have been reported to activate it in yeast (Uluisik et al. 2011; Narotsky et al. 1998; WHO 1998). Here we report that treatment of DU-145 prostate cancer cells with physiological concentrations of BA causes mild ER stress with ER expansion, the formation of cytoplasmic stress granules, and activation of the eIF2α/ATF4 integrated stress response (ISR).

## Materials and methods

### Chemicals

Boric acid, Tris, NaCl, MgCl_2_, sucrose, DTT, methanol, and DMSO were purchased from Sigma-Aldrich (St. Louis, MO). Glutaraldehyde, cacodylic acid, lead citrate, and uranyl acetate were purchased from Electron Microscopy Supplies (Hatfield, PA). Paraformaldehyde was purchased from Affymetrix/USB Corporation (Cleveland, OH). TritonX-100, Tween-20, NP40, and cycloheximide were purchased from Fisher Scientific (Pittsburg, PA). BSA and thapsigargin were purchased from Santa Cruz Biotechnologies (Santa Cruz, CA). Fetal Bovine Serum (FBS) was purchased from Gibco-Life Sciences (Grand Island, NY). Protease and phosphatase inhibitors were purchased from Calbiochem (San Diego, CA).

### Cell culture

DU-145 prostate cancer cells, obtained from the American Type Culture Collection (Manassas, VA), were maintained in RPMI Media 1640 (Gibco-Life Technologies, Grand Island, NY) supplemented with 10 % FBS, penicillin (100 U/ml), streptomycin (100 µg/ml), and l-glutamine (200 mM) (Gemini Bio-Products, Sacramento, CA). Cells were plated on 10 or 15 cm plates (Corning Life Sciences, Corning, NY) and incubated at 37 °C in a humidified chamber containing 5 % CO_2_ and 95 % air and grown to 80 % confluency. Our procedure to remove boron from media has been previously described (Bennett et al. 1999). All treatment groups in the present study used media that had been stripped of boron by shaking with 2 g of Amberlite IRA 743 exchange resin (Sigma-Aldrich) for 12 h at 4 °C.

### Transmission electron microscopy (TEM)

Cells were grown on plastic cover slips (Nalgene, Rochester, NY) to no more than 80 % confluency and treated with BA-supplemented complete media at concentrations of 0, 10, 50, and 250 µM for 24 h, followed by fixation with 2 % glutaraldehyde in 0.1 M sodium cacodylate buffer. Samples were dehydrated with increasing concentrations of ethanol followed by infiltration and embedding in RL White acrylic resin (Ted Pella, Redding, CA). The resin embedded cells were sectioned in 100 nm slices and placed on copper grids. The sections were counter stained with 2 % aqueous uranyl acetate at 57 °C for 1 h followed by 4 % lead citrate staining for 1 min. Imaging by TEM was performed using a JEOL 100CX transmission electron microscope.

### Immunofluorescent microscopy

DU-145 cells were grown to 80 % confluency on glass cover slips (Fisher Scientific, Pittsburg, PA) and treated with either BA-free media (0), BA-free media supplemented with BA, or 1 μM thapsigargin. Cells stained for TIA-1 were first fixed with 4 % paraformaldehyde in PBS and permeabilized with 0.5 % TritonX-100 in PBS. Fixed cells were blocked with 10 % FBS in PBS overnight. Next, they were incubated in a humidity chamber with anti-TIA-1 (Santa Cruz Biotechnology) monoclonal antibody at concentrations of 1:50 followed by secondary FITC at 1:100. Coverslips were mounted with a mixture of Vectashield with 4′,6-diamidino-2-phenylindole (Vector Laboratories, Burlingame, CA) and regular HardSet Vectashield (Vector Laboratories) mounting mediums at 1:5 respectively. Images were captured with an Olympus DP72 camera (Olympus America, Center Valley, PA) connected to an Olympus BX51 fluorescence microscope (Olympus America) using an Olympus UIS2 UPlanFLN 100X/1.30 OilPh3 objective (Olympus America) and FITC and DAPI filters. Either Olympus DP2-BSW (Olympus America) or Adobe Photoshop (Adobe Systems Incorporated, San Jose, CA) software was used to merge and crop images.

### Immunoblot analysis

DU-145 cells grown on 15 cm plates (Corning) to 80 % confluency were treated with BA-free media (0) or BA-free media supplemented with BA, 1 µM thapsigargin, or DMSO positive control vehicle for varying time points. Cells were washed with ice cold phosphate buffer solution (PBS) supplemented with 0.1 % Tween (PBST) prior to adding 100 µl radio immuno-precipitation assay (RIPA) lysis buffer. Cell lysates were scraped from plates using a spatula (Corning) and passed through a 23 gauge needle (BD, Franklin Lakes, NJ) 8–10 times on ice. The protein was quantitated using the Coomassie Plus Protein Assay (Thermo-Scientific, Waltham, MA). Aliquots of 30–35 µg protein were run on a 4–12 % gradient TGX SDS-PAGE (Bio-Rad, Hercules, CA) at 200 V for 30 min along with a molecular weight ladder (Bio-Rad). Protein was transferred to a nitrocellulose membrane in transfer buffer with 20 % methanol at 40 V for 1.5 h. Membranes were blocked in 3 % BSA with 37.5 mM Tris (pH 8.8), 125 mM NaCl, and 0.1 % Tween 20 for at least 4 h. Following blocking, the membranes were incubated with the primary antibody for 1 h in PBST or 3 % BSA blocking solution, washed in PBST, and incubated with the appropriate secondary antibody with a HRP tag, followed by washing three times with PBST. The membranes were exposed to ECL Plus (Amersham/GE Healthcare, Pittsburg, PA) for 2–5 min and imaged using a Typhoon 9410 Variable Mode Imager (Amersham). Densitometry was performed using ImageQuant 5.2 software (Molecular Dynamics, Pittsburg, PA). All secondary antibodies were purchased from Santa Cruz Biotechnologies (Santa Cruz, CA). The following primary antibodies from Santa Cruz Biotechnology were used: TIA-1 (goat polyclonal), Grp78/BiP (mouse monoclonal), Actin (goat polyclonal), GAPDH (mouse monoclonal) and ATF4 (rabbit polyclonal). The eIF2α (rabbit polyclonal) and ph-eIF2α (rabbit polyclonal) were purchased from Cell Signaling (Danvers, MA).

### Taqman real time PCR (RT-PCR)

DU-145 cells were grown on 10 cm plates (Corning) to 80 % confluency at least 24 h prior to treatment. Cells were treated with BA and analyzed at various time points. RNA was isolated from cells using an RNeasy mini kit (Qiagen, Valencia, CA). Total RNA (2 µg) was reverse transcribed using Superscript III Reverse Transcriptase (Invitrogen) with random hexamer primers (Life Technologies) at a final volume of 20 µl at 25 °C, 10 min (10:00); 50 °C, 45:00; and 70 °C, 15:00. Applied Biosystems (ABI, Foster City, CA) Taqman predesigned assays were used for all genes as well as GAPDH as a control internal housekeeping gene. Plates were read by a 7500 Fast Real Time PCR System using the 7500 Fast System Software v1.4.0 (ABI). Quantitation of gene expression levels were calculated from a standard curve created from reactions containing a combination of cDNA from all treatments for each gene.

### Cell cycle analysis

Flow cytometry was performed to determine if BA caused a shift in cell cycle to the G1 phase. DU-145 cells were treated with 0 and 50 µM BA for 24 h. The cells were washed with PBS, scraped from the plate with a rubber policeman, centrifuged for 5 min at 1,200 rpm, and resuspended in 1 ml of DNA staining hypotonic propidium iodide solution (1 mg/ml sodium citrate, 0.3 % tritonX100, 100 µg/ml propidium iodide, 20 µg/ml ribonuclease A). The cells were incubated for 30 min followed by cell cycle analysis using a FACS Calibur Flow Cytometer with Modfit LT 3.0 software for data analysis. Analysis of each sample was performed on greater than 10,000 events and coefficients of variation less than 5 %.

### Statistical analysis

All immunoblot were analyzed using the unpaired Student’s *t* test. All time points represent 3–6 replicates. ImageJ software (NIH, Bethesda, MD) was used for immunofluorescence quantitation. Cells with clear borders between the nucleus and cytoplasm were selected for analysis. The intensity of areas of equal size in the nucleus and cytoplasm was quantified using the histogram tool in RGB mode. RT-PCR data points represent 3–6 replicates and were analyzed using the unpaired Student’s *t*-test.

## Results

### BA causes dose-dependent changes in ER morphology

**Fig. 1:**
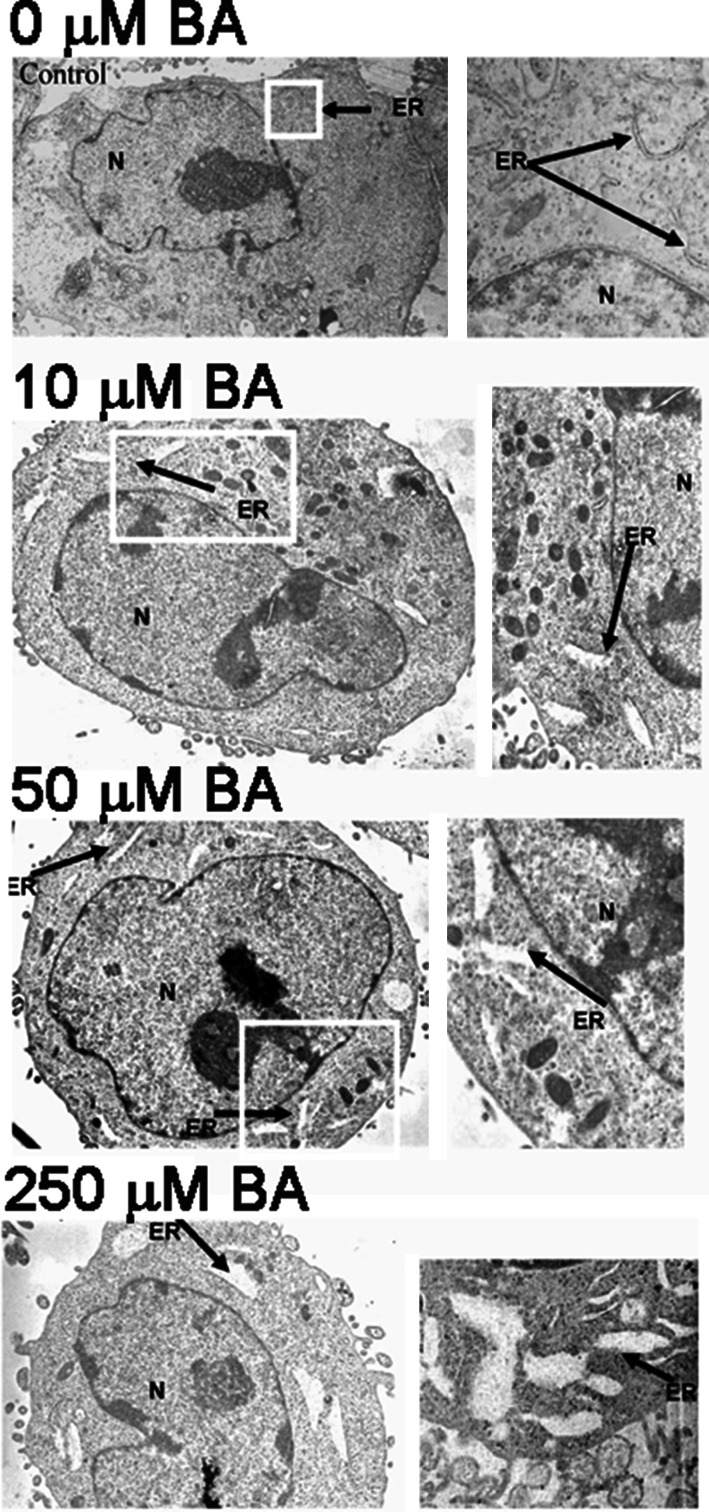
BA induces ER vacuolization and expansion in DU-145 cells. TEM images of DU-145 cells treated for 24 h with 0, 10, 50, and 250 µM BA (n = 3). Cells exhibited a dose-dependent swelling and vacuolization of the ER, (*N* nucleus, *ER* endoplasmic reticulum). *White boxes* indicate magnified area shown on the *right*

Light microscopy analysis of BA treated DU-145 cells showed cell flattening (Barranco and Eckhert 2006), therefore we were interested in knowing how this morphological change manifested at the ultrastructural level. We used TEM on DU-145 cells treated for 24 h with 0, 10, 50 or 250 µM BA. The results show a dose-dependent increase in ER expansion, cytoplasmic granularity and cytoplasmic vacuole formation (Fig. 1).

### BA induces phosphorylation of eIF2α and the formation of cytosolic stress granules

**Fig. 2:**
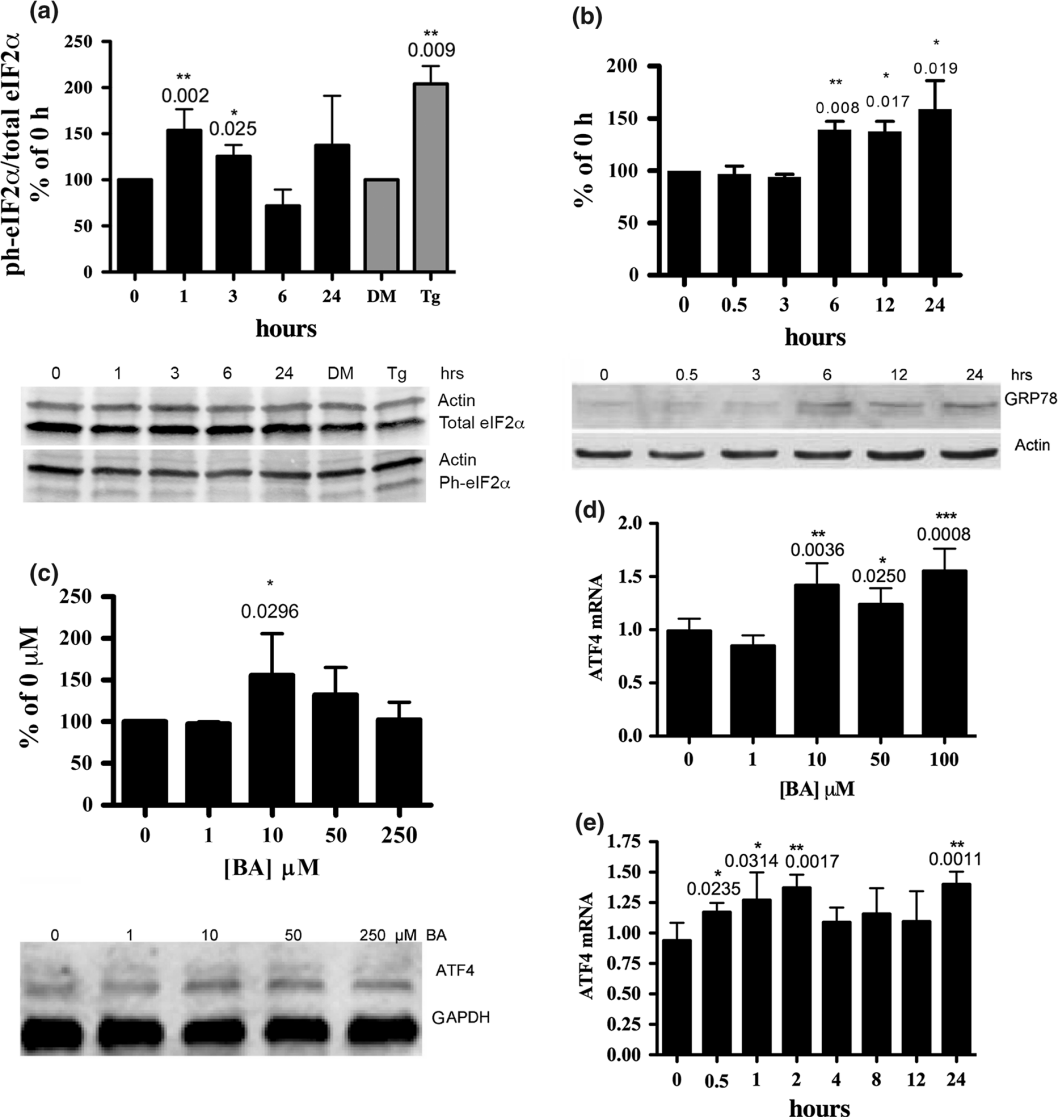
BA induces the eIF2α/ATF4 pathway. **a** 50 µM BA treated for 24 h induced phosphorylation of eIF2α in DU-145 cells at 1, and 3 h of treatment (n = 3–5). DU-145 cells treated with 1 µM thapsigargin (Tg) or DMSO (DM) for 1 h, n = 3. **b** DU-145 cells treated with 50 µM BA for 0, 0.5, 3, 6, 12 and 24 h. GAPDH was used as an internal loading control. Translation of GRP78/BiP increased in cells treated at 6–24 h, (n = 3–4). **c** Treatment of DU-145 cells with 10 µM BA for 24 h caused a significant increase in ATF4. **d** DU-145 cells were treated with different doses of BA for 24 h and ATF4 mRNA levels measured using real-time PCR (n = 3–6). Significant increases in mRNA were observed at 10, 50 and 100 μM BA. **e** A time course study following treatment of DU-145 cells with 10 μM BA showed significantly higher ATF4 mRNA levels at 0.5, 1, 2, and 24 h (n = 3–6). Significance differences from 0 concentration or time are shown and represented using *p < 0.05, **p < 0.01 and ***p < 0.001. *Error bars* represent standard deviations

Cells respond to changes in their environment by reprogramming translation to adapt to the new conditions (Kawai et al. 2004). eIF2α is the regulatory subunit of the large ternary complex, eIF2–GTP–tRNAi Met, which positions the initiator methionine at the first codon of mRNA to commence translation and protein synthesis. Phosphorylation of eIF2α on serine 51 inhibits the formation of the complex, thus inhibiting global translation allowing cells to recover from ER stress (Zoll et al. 2002). We observed an increase in phosphorylation of eIF2α from 1 to 3 h following treatment of DU-145 cells with 50 µM BA (Fig. 2a). We used thapsigargin as positive control since it induces ER stress by decreasing ER luminal Ca^2+^. Thapsigargin also significantly activated phosphorylation of eIF2α in DU-145 cells at 24 h (Fig. 2a).

**Fig. 3:**
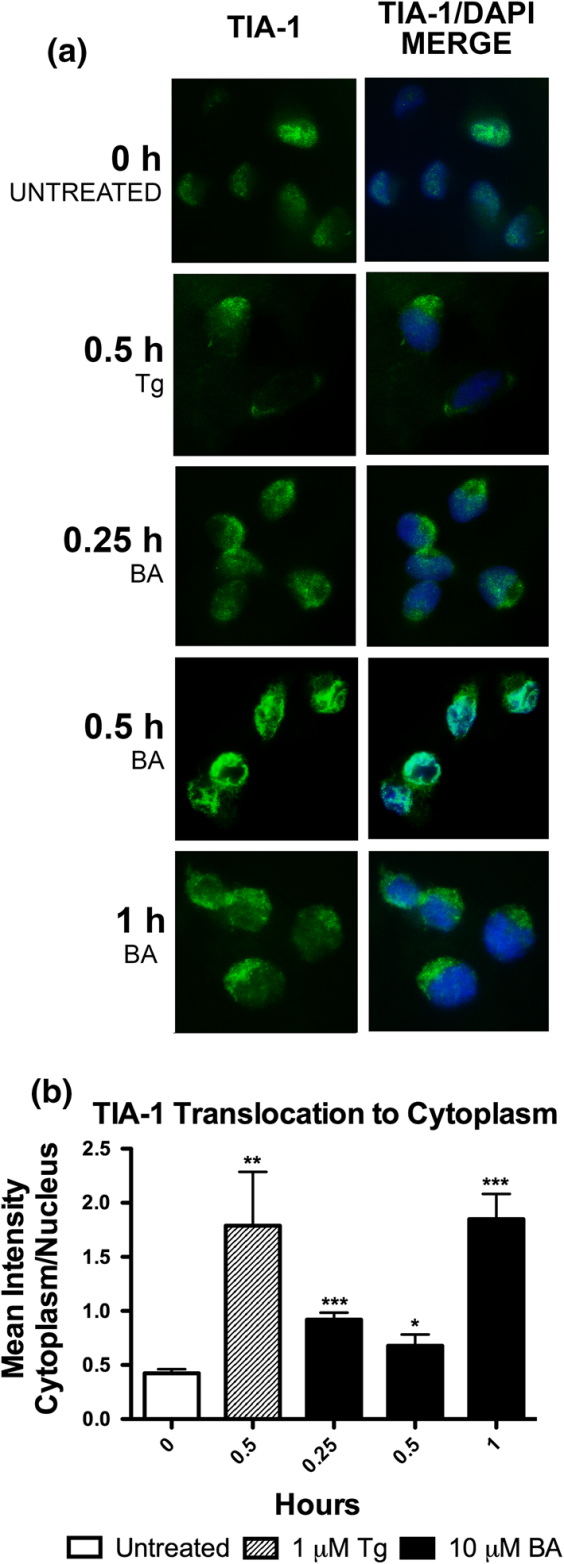
BA induces formation of TIA-1 positive stress granules. 10 µM BA induced formation of TIA-1 positive stress granules in DU-145 cells at 0.25, 0.5, and 1 h, indicated by TIA-1 protein (*green*) moving out of the nucleus (*blue*) with BA treatment. 1 µM thapsigargin (Tg) was used as a positive control, (n = 6–24 cells). All time points showed a significant increase. Pictures are representative of results. Significance differences were represented as *p < 0.05, **p < 0.01, and ***p < 0.001 and *error bars* represent standard deviations. (Color figure online)

The absence of eIF2–GTP–tRNAi Met results in eIF2/eIF5-deficient, ‘stalled’ 48S pre-initiation complex (Kedersha et al. 2002). The eIF2/eIF5-deficient pre-initiation complexes and their associated mRNA transcripts are routed to cytoplasmic stress granules (SG) in a process that requires the RNA-binding proteins T-cell intracellular antigen-1 (TIA-1) and TIAR (Anderson and Kedersha 2002). TIA-1 normally resides in the nucleus of unstressed cells but under stress conditions is recruited into SGs. Nearly 90 % of TIA-1 and about 50 % of cytoplasmic poly(A) RNA and poly(A)-binding protein-1 is recruited into SGs (Anderson and Kedersha 2002; Kedersha et al. 2002). We treated cells with 10 µM BA or thapsigargin as a positive control and used a polyclonal antibody to TIA-1 to positively identify cytoplasmic granules as SG. TIA-1 translocation from the nucleus began to occur 15 min after treatment and reached a maximum by 1 h (Fig. 3). This correlated with the timing of 1 h maximum for eIF2α phosphorylation (Fig. 2a).

### BA treatment increases GRP78/BiP

Glucose-regulated protein binds to and maintains the transmembrane sensor PERK in an inactive form (Hendershot 2004; Pfaffenbach and Lee 2011). GRP78/BiP protein levels significantly increased in DU-145 cells treated with 50 µM BA for 6, 12 and 24 h post-treatment (Fig. 2b).

### BA induces ATF4

Phosphorylation of eIF2α activates synthesis of ATF4, a transcription factor in the highly conserved ISR that enables cells to adapt to changing environmental conditions. We used polyclonal antibodies to measure ATF4 protein and observed an increase at 24 h in cells treated with 10 µM BA (Fig. 2c). The results of a dose response study showed the expression of ATF4 mRNA was increased by treatment with 10, 50 and 100 µM BA, but not 1 µM BA (Fig. 2d). A time course study showed the expression of ATF4 mRNA in cells treated with 10 µM BA was higher at 30 min, 1 and 2 h post treatment (Fig. 2e). ATF4 mRNA levels returned to normal in the interval between 4 and 12 h and increased again at 24 h.

### Cell cycle percentages were not affected by a 24 h treatment of BA

**Fig. 4:**
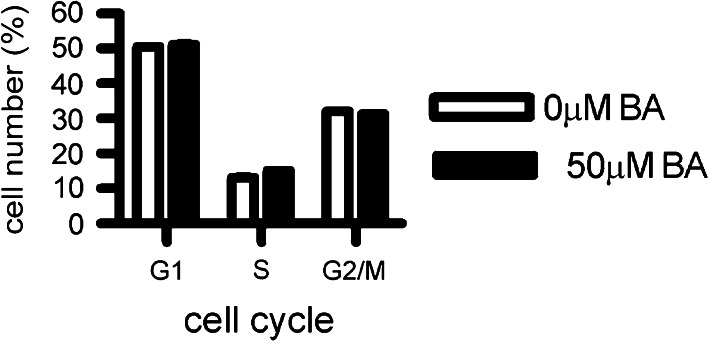
The cell cycle was not significantly altered by BA treatment. Flow cytometry analysis was conducted a 24 h treatment with 0 or 50 µM BA. The percent of cells treated with 0 or 50 µM BA were: G1 phase 50.59 ± 0.75 and 51.21 ± 0.62 %, S phase 13.09 ± 1.16 and 15.15 ± 0.38 %, G2 phase 32.09 ± 0.46 and 31.53 ± 0.18 % respectively, (n = 3). The 2 % shift in S phase was of doubtful biological significance. Standard deviation *error bars* were plotted, but are too close to the *bar graph* to visualize

Flow cytometry analysis following a 24 h treatment with 50 µM BA had little impact on the cell cycle. The percent of cells treated with 0 or 50 µM BA were: G1 phase 50.59 ± 0.75 and 51.21 ± 0.62 %, S phase 13.09 ± 1.16 and 15.15 ± 0.38 %, G2 phase 32.09 ± 0.46 and 31.53 ± 0.18 % respectively (Fig. 4). The 2 % shift in S phase (p value = 0.03) was so small it was doubtful it had biological meaning. This result is consistent with previous reports demonstrating that BA does not cause a biologically significant effect on the cell cycle (Barranco and Eckhert 2004; Bradke et al. 2008). We did not observe an apoptotic cell population after BA treatment.

## Discussion

A review conducted by the Institute of Medicine of the available data on dietary boron concluded it was beneficial but the molecular mechanisms underpinning its positive effects were unknown (IOM 2001). We describe in this article molecular responses of DU-145 human prostate cancer cells that follow treatment with physiological concentrations of BA. We draw several conclusions from our experimental results.

First, BA causes a dose- and time-dependent expansion of the ER with formation of cytoplasmic SGs (Figs. 1, 2). We positively identified cytosolic granules as SGs using an antibody to the tumor suppressor TIA-1, a classic biomarker of SG formation (Kedersha et al. 1999). TIA-1, with TIAR, act downstream of ph-eIF2α to promote the recruitment of untranslated mRNAs into SGs which are essential in regulating developmental and stress-responsive pathways at the level of alternative pre-mRNA splicing and mRNA translation (Kedersha et al. 1999; Buchan and Parker 2009; Bauer et al. 2012). This allows for temporary confinement of mRNA transcripts and reduces their ability to interact with other molecules while preventing their degradation (Buchan and Parker 2009). Activation of TIA-1 in HeLa cells has been shown to prevent cell proliferation, tumor growth, and invasion (Izquierdo et al. 2011). Our observation that BA activates TIA-1 in DU-145 cells suggests this is part of the mechanism that underpins BA’s chemopreventive and biological activity. The ability of BA to activate TIA-1 provides insight into a potential mechanism underlying BA stimulated increases in TNFα in human fiberblasts and pigs (Armstrong and Spears 2003; Benderdour et al. 1998). TIA-1 is a component of a regulatory complex that binds to an adenine/uridine-rich element in the 3′UTR of TNFα mRNA. TIA-1 binding represses TNFα mRNA translation under non-stress conditions, but releases it during ER stress (Gueydan et al. 1999; Piecyk et al. 2000). Our results suggest BA may induce TNFα by causing TIA-1 to translocate from the nucleus to stress granules in the cytoplasm, thus removing its repressive effect (Benderdour et al. 1998; Armstrong and Spears 2003).

Second, BA increases eIF2α phosphorylation and BiP/GRP78 translation. BiP/GRP78 inactivates PERK and is the major ER Ca^2+^ storage protein and is regulated by luminal Ca^2+^ levels (Lievremont et al. 1997). BIP/GRP78 is released and PERK is activated and phosphorylates eIF2α when luminal Ca^2+^ levels fall, (Lievremont et al. 1997). Our results show phosphorylation of eIF2α is a time-dependent response that peaks 1 h post-treatment which is within the time frame reported for BA to decrease ER Ca^2+^ in DU-145 cells (Henderson et al. 2009).

Third, BA upregulates the transcription factor, ATF4. Phosphorylation of eIF2α increases upstream open reading frame (uORF) mediated translation of bone related activating transcription factor 4 (ATF4)(Vattem and Wek 2004). The ph-eIF2α/ATF4 pathway is highly conserved from yeast to mammals and has been named the ISR because it is a target of many different types of environmental stresses (Harding et al. 2003). Our observation that BA induces mild activation of ATF4 in prostate cancer cells may provide insight for understanding how boron supplementation increases bone mass and strength (Hunt 1996; Armstrong and Spears 2003; Nielsen and Stoecker 2009). Mild activation of ATF4 regulates osteogenesis during development and postnatally regulates bone remodeling and the osteoblast specific gene osteocalcin (Wang et al. 2012). Parathyroid hormone mediates its functions in part by regulating binding of ATF4 to osteocalcin which is an essential regulator of endochondral bone formation and for the reversal of bone loss and (Danciuk et al. 2012).

In summary, previous work has shown that BA is a competitive inhibitor of cADPR induced Ca^2+^ release from the ryanodine receptor, and decreases ER Ca^+2^ (Henderson et al. 2009). In this paper we demonstrate that mild ER stress and activation of the ISR, both known to be initiated by ER Ca^2+^ depletion, occur following treatment of DU-145 prostate cancer cells with physiological concentrations of BA. In the context of the literature, our present findings suggest mild ER stress with mild activation of the eIF2α/ATF pathway may underpin the anticarcinogenic and bone strengthening effects of boron.

## Abbreviations

BA: Boric acid
cADPR: Cyclic ADP ribose
ER: Endoplasmic reticulum
TIA-1: T-cell intracellular antigen-1
Grp78: Binding immunoglobulin protein (BiP) also known as 78 kDa glucose-regulated protein
ATF4: Activating transcription factor 4
SG: Stress granule
ISR: Integrated stress response
